# The effects of mindfulness upbringing perception on social entrepreneurship orientation: A moderated mediation model of prosocial motivation and perceived pressure from external stakeholders

**DOI:** 10.3389/fpsyg.2022.968484

**Published:** 2022-10-13

**Authors:** Tingting Shan, Xiaoya Tian

**Affiliations:** School of Management, Nanjing University of Posts and Telecommunications, Nanjing, China

**Keywords:** mindfulness upbringing, prosocial motivation, social entrepreneurship orientation, perceived pressure from external stakeholders, a moderated mediation model

## Abstract

Driven by economic and social benefits, social enterprises create new development models that combine wealth creation, social welfare provision, and environmental improvement through innovative approaches. The social entrepreneurship orientation reflects the behavioral tendency to transplant entrepreneurship orientation into the field of social value creation. It is a strategy to balance and integrate economic interests and social interests, which has a significant impact on social entrepreneurship performance. The purpose of this study is to explore the internal mechanism of the impact of social entrepreneurs’ mindfulness upbringing perception on social entrepreneur orientation. To reveal the internal mechanism, we propose a moderated and mediation model of prosocial motivation and perceived pressure from external stakeholders. In this study, random sampling was conducted among social start-ups in China. In order to improve the accuracy of the scale, a pre-survey was conducted before the formal survey. The data analysis results of the pre-survey showed that the scale in this study was suitable for the Chinese context and had good external validity. Through using survey data from social entrepreneurs in China, hierarchical regression analysis and bootstrapping model are adapted to test and verify mediation and moderation effects. The results show that mindfulness upbringing perception indeed positively influences social entrepreneurship orientation directly and partly through the mediating effect of prosocial motivation. Moreover, findings suggest the perceived pressure from external stakeholders negatively moderates not only the relationship between prosocial motivation and social entrepreneurship orientation but also the overall mediation model. This indicates that social entrepreneurs with low perceived pressure from external stakeholders will improve their social entrepreneurship orientation rapidly when their prosocial level is high. Based on these findings, we conclude that social entrepreneurship orientation may be achieved more effectively through the complex process of mindfulness upbringing perception, prosocial motivation, and perceived pressure from external stakeholders. Finally, the study proposes the theoretical and practical implications and suggestions for follow-up research.

## Introduction

### Theoretical basis and hypothesis

#### Mindfulness upbringing perception

Family upbringing refers to a series of activities in which parents create the desired emotional atmosphere around their children in the process of child-rearing, which is embodied in parents’ attitude, behavior, and non-verbal elements towards their children (Jean et al., 2016; He et al., 2019; Gauthier et al., 2021). There are different types of parenting (Sedikides et al., 2015). In the most classic classification method, Baumrind (1966) and Maccoby and Martin (1983) defined four types according to parents’ “requirements” and “responses” to their children: authoritarian, authoritative, permissive, and neglective. In recent years, the taxonomy of family upbringing has received renewed attention, and “new” types have been conceptualized, such as strict upbringing that reflects parents’ attitudes towards their children (Hoover et al., 2022), narcissistic parenting focusing on parental psychological experience (Evans et al., 2018), and overparenting examining the degree of monitoring of children (Duff, 2022). Different parenting styles determine the level of parenting mindfulness. The characteristics of Chinese family upbringing have also received attention in recent years. For example, Liu et al. (2011) studied the problem of overparenting in China; Adcock et al. (2021) believe that the parenting style advocated in traditional Chinese culture is similar to authoritarian parenting. That is, parents tend to respond negatively to the needs of their children, emphasize expectations on their children and require them to abide by rules strictly. In addition, Tseng and Reeve (2011) comparative study found that Chinese parents in The United States were more autocratic than European parents in terms of upbringing.

Interestingly, however, children of Chinese descent were more likely than children of European descent to think their parents’ punishment was fair (Lidia et al., 2022). These results suggest that Chinese parenting and its functions have subtle characteristics different from those of the West. The level of parenting mindfulness varies with different parenting styles (Egan et al., 2022). In the context of Chinese culture, most social entrepreneurs mentioned in the interview that their perception of family mindfulness upbringing in childhood influenced their career choice (Hirshberg et al., 2022). Although they and their parents did not know what mindfulness upbringing was then, the parenting style their parents gave them when they were young more or less affected their entrepreneurial motivation in the future. Therefore, mindfulness upbringing plays a crucial role in social entrepreneurship orientation, but the complex internal mechanism is not very clear (Beauchaine et al., 2013; Ishak et al., 2015).

Mindfulness parenting was proposed by Kabat-Zinn and other scholars in the 1980s. It extends mindfulness theory and mindfulness therapy in Zen and psychology in family education. It refers to parents’ conscious and non-judgmental attention and awareness of their own and their children’s internal state and the interaction process of parenting in the context of this moment (Tellez Infantes et al., 2022). Mindfulness upbringing perception affects not only the health and life of children, such as health risk motivation and lifestyles (Juul et al., 2022), extroversion, and prosocial motivation but also their work and career, including the formation of adolescent human capital, children’s sense of occupational efficacy, occupational adaptability (Ohtsubo and Watanabe, 2013), and even attitudes to change (Imas, 2014). One reason why mindfulness upbringing perception influences children’s social entrepreneurship orientation may lie in the similarities between social entrepreneurship and parenting, such as the emphasis on vision and strategy, leading by example, two-way communication, trust and integrity, and the realization of social values (Mary-Hunter and Brayden, 2019). These intersections make parents naturally become role models in developing their children’s social entrepreneurship orientation. According to social learning theory (Frolli et al., 2021), the concept of role model is helpful in understanding the formation of social entrepreneurship orientation: social entrepreneurship behavior is the result of socialization -- in socialization, parents have relatively close and frequent contact with them, thus becoming an important role model of social entrepreneurship orientation for the latter (Dunfield and Kuhlmeier, 2013). Specifically, in the process of mindfulness upbringing perception influencing the development of social entrepreneurship orientation, the actual bridge may include not only parents’ attitudes and behaviors but also children’s reactions (Michael and Cunningham., 1988; Stephan, 2011; Hair et al., 2013; Barker and Lori, 2021; Frank et al., 2021; Nascan et al., 2021). External stress is one of the most apparent constraining factors -- increasing external stress diminishes the effect of mindfulness upbringing perception. However, this is not so much a function of external pressure as a reflection of individual initiative (Li, 2021). Amodu and Ama (2016) proposed reflected modeling to explain the above phenomenon: with the increase of external pressure, individuals will be exposed to the role model of social entrepreneurship orientation, such as external stakeholders, and then reflect on the value of parents as the role model of social entrepreneurship orientation (Ramus and Killmer, 2010). Therefore, the development of social entrepreneurship orientation results from individual integration of various role models of social entrepreneurship orientation (Kraus et al., 2017; Zaninotto et al., 2022).

#### Social entrepreneurship orientation

Social entrepreneurship is driven by various social issues (wealth gap, aging, environmental protection) that accompany global economic development (Kallmuenzer and Peters, 2017). Compared with commercial enterprises, social enterprises focus on creating social value, while with charitable organizations and non-profit organizations, social enterprises have a specific economic value creation function, which can be used to subsidize philanthropic donations and government subsidies (Nandamuri and Gowthami, 2015). Social entrepreneurs play a decisive leadership role in social entrepreneurship (Alex, 2014; Gali et al., 2020). Driven by a vital social mission, they are skilled at identifying and discovering social problems and business solutions that others cannot find to provide public services (Sengupta and Sahay, 2017). Through active social entrepreneurship, they meet diverse social needs in various fields and help the government solve multiple social problems, such as unemployment, urban–rural gap, unfair distribution, imperfect social security system, and environmental damage (Gauthier et al., 2021). Driven by the combination of economic and social benefits, social enterprises have established new development models through innovative ways that combine the functions of creating wealth, providing social welfare, and improving the environment (Shin and Park, 2019). Social entrepreneurship is double-oriented in terms of competition and public interest. According to Dees’ view of the continuum spectrum of social entrepreneurship, dual orientation is a relationship of “one body and two sides,” which realizes complementarity, adjustment and dynamic balance in the development process based on situational changes (Freiling et al., 2014). Although public-interest orientation (social value) is the ultimate goal, competitive orientation (economic means) is indispensable, which is an important prerequisite for social entrepreneurship to realize self-management, and self-financing (Dwivedi and Weerawardena, 2018). The two complement each other and jointly promote the sustainable development of social entrepreneurship, which is also an essential reason why social entrepreneurship is different from traditional public welfare and philanthropy (Zafar et al., 2022).

In entrepreneurial activities, economic goods and social values should shift from “conflict” to “integration” (Calic and Mosakowski, 2016). Self-interest-driven entrepreneurship that damages social interests should be restrained to guide the improvement of entrepreneurial quality (Moreno and Casillas, 2010). Social entrepreneurship can be regarded as entrepreneurship guided by social goals. At the same time, the positive effects of social entrepreneurship on inclusive economic development can be seen as changing the internal thinking mode of entrepreneurial activities and inspiring entrepreneurs to become more thoughtful. Social entrepreneurship orientation developed from entrepreneurship orientation (Devi et al., 2015). Entrepreneurial orientation, derived from strategic choice theory, refers to activities related to firm behavior, decision making, and organizational process. Social entrepreneurship orientation reflects the behavioral tendency to transplant entrepreneurship orientation into the field of social value creation (Cassia et al., 2014). In the face of such a complex variety of social needs and social problems, as well as the lack of natural resources for public welfare undertakings, it is a topic of great practical significance for social entrepreneurs to make entrepreneurial decisions and form social solid influence by choosing the entrepreneurial’s tendency with both profit and social “double bottom line” (Talebi et al., 2015). It is of great significance to identify the pre-influencing factors of social entrepreneurship tendency to promote the performance growth of social enterprises, solve social criticism, and break the welfare deadlock and promote the sustainable development of society (Brndle et al., 2019). This is also the necessity of this study.

#### Mediating effect of prosocial motivation

Motivation refers to a desire or reason to act, and “prosocial” literally means an intention to help or benefit another person (Bardacke and Duncan, 2014; Mohammad et al., 2018). The prosocial motivation of social entrepreneurs is the desire to benefit other people or groups through social entrepreneurship activities. To understand prosocial structure more deeply, it is necessary to place the viewpoint of prosocial motivation in the basic framework of motivation (Autera, 2015). Psychologists believe that motivation has three levels of universality: global, situational, and episodic. The scope of these three levels decreases, and the constraint conditions increase (Sarbandi et al., 2015). International motivation focuses on the relatively stable personality orientation of social enterprise entrepreneurs, with specific goals and actions across time and situations. Situational motivation focuses on the motivation of social enterprise entrepreneurs for a particular field or category of behavior and changes moderately in time and context. Episodic refers to the highly variable motivation of social enterprise entrepreneurs for a specific behavior at a particular time (Warriner et al., 2018). Therefore, in extreme cases, global motivation can be regarded as a personal trait inherent in the entrepreneur, while situational motivation and episodic motivation are more of a flexible ability and tendency to adapt to change. In response, the prosocial motivation of social entrepreneurs can be divided into three dimensions (Duncan and Shaddix, 2015). Global prosocial motivation refers to the tendency of social enterprise entrepreneurs to care about the interests of others and try to protect and promote the well-being of others through social entrepreneurship activities (Rodríguez-Meirinhos et al., 2021). The situational prosocial motivation of social entrepreneurs refers to the desire of social enterprise entrepreneurs to benefit other people of a specific category through a specific field, operation process, or business model (Davis et al., 2014). Entrepreneurial behaviors under situational prosocial motivation include a car wash business for unemployed mentally disabled people and a handicraft business for rural women with low education levels. Episodic prosocial motivation refers to the desire of social enterprise entrepreneurs to benefit others in a particular group in a particular situation (Abatemarco, 2014). For example, going back to the previous models, one social entrepreneur started a car wash business for unemployed mentally disabled people in western China, and another social entrepreneur wanted to create a handicraft business for poorly educated women in the economically underdeveloped west and central regions of China, etc.

According to the emotional contagion theory, entrepreneurs who receive mindfulness training in the early years tend to form organizational ethics and paradoxical leadership within the organization and are good at creating a loose and harmonious environment within the organization, and creating an atmosphere and awareness of social entrepreneurship in the organization (Gannon, 2015). At this time, if they face the support of resources, it will undoubtedly strengthen the intensity of social entrepreneurship (Bang et al., 2021). Thus, the competitive and public-welfare-oriented social entrepreneurship strategy can be effectively triggered. Therefore, this study proposes the following hypotheses:

*Hypothesis1*: Mindfulness upbringing perception has a positive impact on social entrepreneurship orientation.

*H1a*: Mindfulness upbringing perception positively influences competitive orientation.

*H1b*: Mindfulness upbringing perception positively influences public-welfare orientation.

According to self-determination theory, prosocial motivation is introspective, result-oriented, and future-oriented (Ying and Wang, 2019; Garon et al., 2021). Family upbringing plays a vital role in the intergenerational transmission of family cultural capital and the cultivation of children’s social communication ability and positive psychological quality (Sulphey and Salim, 2020; Moussaoui Lisa et al., 2022). Social entrepreneurs who received full mindfulness training in the early stage generally expressed those parents can timely and acutely perceive and respond to the needs of their children, give them adequate care, support and understanding, and encourage the cultivation of independent ability (Medeiros et al., 2015). Based on the self-determination theory, this study explores the effects of mindful upbringing perception on entrepreneurial motivation from the perspective of motivation synergy. It introduces prosocial motivation into the mechanism of the interaction between mindful upbringing perception and social entrepreneurship motivation (Lee et al., 2021). Mindful parenting perception stimulates prosocial motivation in social entrepreneurs. This action path is mainly realized through the following mechanisms: First of all, the parent–child relationship is closer under the mindful parenting style, the children’s negative emotional experience of insecurity is significantly reduced, and it is easier to establish a trusting relationship with others, thus strengthening the prosocial motivation of the children (Lin and Desai, 2022). Secondly, under the mindfulness parenting style, when parents meet their children’s needs for independent development, children are more inclined to actively think and master strategies to deal with difficulties, and then improve their sense of self-efficacy. It also encourages children to have a strong sense of responsibility and emotional regulation ability in the face of complex tasks, and to acquire effective coping styles and solutions (Kohut et al., 2016). And then develop a high level of self-efficacy and environmental control, forming a solid pro-social motivation (Gunilla et al., 2018). Thirdly, the stronger the perception of mindful parenting, the easier it is to cultivate and accumulate resilience and strengthen prosocial motivation (Hooi et al., 2016; Purevdulam, 2017). These arguments suggest the following hypothesis:

*Hypothesis2*: Mindfulness upbringing perception is positively associated with prosocial motivation.

According to the resource dependence theory, the prosocial motivation of entrepreneurs brings the harmonious relationship between enterprises and customers, suppliers and competitors, which will undoubtedly trigger enterprises to develop new products and services in a forward-looking manner, so as to perceive market changes in advance and take advanced actions to achieve better market performance (He, 2018). In addition, individuals with prosocial motivation will consider the interests of others in organizational activities. They will bring more information and knowledge sharing inside and outside the organization, which intangibly intensifying the competitive orientation. Therefore, prosocial motivation has a positive impact on the competitive direction of enterprise social entrepreneurship (Gordon and Chapman, 2018). According to the motivational information processing theory, prosocial motivation helps individuals jump out of the limitations of their perspective, improve their sensitivity to others’ views and needs, enhance their ability of perspective-taking and viewpoint integration, and generate positive emotions to enhance the level of creativity (Bruin et al., 2014). Social entrepreneurial enterprises are more inclined to absorb external heterogeneous knowledge, promote cross-border search and opportunity identification, and establish their competitive advantages. They are more prone to be aggressive and achieve better performance in the market, which is the essence of competition orientation (Meamar et al., 2016). According to social network theory, prosocial traits help social entrepreneurs form political connections, technological connections, business connections, and other social capital to establish a win-win mechanism among government, market, and the environment through prosocial motivation (Burgdorf et al., 2019). Legitimacy is enhanced, optimal uniqueness is acquired, and the enterprise is driven to build competitive advantage and grow (Gampelaere et al., 2018). Therefore, prosocial motivation can positively predict the competitive orientation of social entrepreneurship.

According to self-determination theory, when social enterprise entrepreneurs perceive that the enterprise fulfills its social responsibility and attaches importance to external stakeholders, they can improve the public welfare orientation of social entrepreneurship from top to bottom through emotional identification and consistency of values (Gl et al., 2020). Unlike prosocial behavior, prosocial motivation refers to the willingness to consider the interests of others and to devote energy to them. According to empathy theory, entrepreneurs with strong prosocial motivation are better able to identify gaps in the market. In the context of social entrepreneurship, prosocial motivation enables entrepreneurs to be highly sensitive to other people’s views and needs, have positive emotions, perspective-taking and dedication consciousness, which will make entrepreneurial activities of enterprises more social orientation, thus triggering enterprises’ social entrepreneurship and public welfare orientation (Gampelaere, 2020). First, individuals driven by prosocial motivation focus on the fairness of outcome distribution. Since individuals consider themselves and others as a whole to combine benefits, harmony and mutual win become the key to cooperation (Caiado et al., 2020). Secondly, social enterprises founded by entrepreneurs with pro-social motivation exhibit ethical characteristics such as fairness, trust and care, which help to build a good and fair working environment and improve the perception of corporate ethics of social enterprise entrepreneurs (Crawford et al., 2015). The prosocial motivation of social entrepreneurs can stimulate organizational loyalty, increase the closeness of work between individuals, and promote initiative, and empathy, helping organizational citizenship behavior of social enterprise entrepreneurs (Gheibi et al., 2020). When individuals have prosocial motivation, on the one hand, they will consider the interests of others more and have more dedication and a sense of mission on an individual basis (Corthorn, 2018). On the other hand, they will devote more time, energy, and wisdom to the organization, resulting in stronger public welfare motivation. Thirdly, motivation information processing theory believes that motivation affects behavior, and the motivation of social enterprise entrepreneurs determines how they process information. According to this theory, when social enterprise entrepreneurs have prosocial motivation, they are more willing to consider problems and obtain information from the perspective of others. They have more willing to cooperate and share information (Burgdorf and Szabó, 2021). Under the influence of prosocial motivation, social enterprise entrepreneurs will produce more positive role behaviors (including in-role behaviors and out-of-role behaviors) in the organization and have stronger social entrepreneurship public-interest orientation compared with entrepreneurs with self-interested motivation. Relevant studies show that the intrinsic work motivation of social enterprise entrepreneurs has a good predictive effect on their positive emotional experience, creative behavior, job persistence, job satisfaction, and social capital accumulation (Grant and Berry, 2011; Alexander, 2017). Individuals with pro-social motivation can redouble their efforts to maximize mutual benefits based on trusting cooperation, which is the essence of social entrepreneurship and public welfare orientation (Rayan and Ahmad, 2017). This study assumes that an entrepreneur can enhance their prosocial motivation to act in the process of the reinforcement of social entrepreneurship motivation, leading to the following hypothesis:

*Hypothesis3*: Prosocial motivation positively influence social entrepreneurship orientation.

*H3a*: Prosocial motivation positively influences competitive orientation.

*H3b*: Prosocial motivation positively influences public-welfare orientation.

Mindfulness upbringing perception may facilitate the enhancement process of prosocial motivation, which leads to social entrepreneurship orientation. These arguments suggest the following hypothesis:

*Hypothesis4*: Prosocial motivation mediating between mindfulness upbringing perception and social entrepreneurship orientation.

*H4a*: prosocial motivation mediating between mindfulness upbringing perception and social entrepreneurship competitive orientation.

*H4b*: Prosocial motivation mediating between mindfulness upbringing perception and social entrepreneurship public-welfare orientation.

#### Moderating effect of perceived pressure from external stakeholders

A moderation effect occurs when there is a third variable between the independent and dependent variables (Chang, 2013; Ahmad, 2016; Gannon et al., 2017). This third variable is called a moderator, which changes the strength or direction of the connection between the two variables. Moderators are generally introduced in previous studies when the relations are inconsistent (Bednall et al., 2013; Shin and Park, 2019). According to earlier investigations, the relation between prosocial motivation and social entrepreneurship orientation appears elusive. Some studies insist that high and intense prosocial motivation enhances the sustained investment in the psychology and behavior of entrepreneurs more effectively (Walker et al., 2020; Aparicio et al., 2021). In contrast, other studies maintain a negative relationship, describing a large firm’s failure to continue entrepreneurship in the emerging market despite having sufficient and high prosocial motivation (Mousavi and Dabiri, 2021). This ambiguous relationship between prosocial motivation and social entrepreneurship orientation suggests the existence of a moderator.

There have been many attempts to confirm the existence of a moderator in fostering and upgrading social entrepreneurship orientation (El-Bassiouny and Letmathe, 2018; Baghestan et al., 2021). The relationship between social entrepreneurship orientation and its determinants or outcomes can be altered not only by endogenous factors, such as personal ability, but also by exogenous elements including the cultural environment in which entrepreneurs grow up (Calam, 2016; Altantsetseg, 2017). Many studies have shown that thinking modes determine behavior patterns (Bardacke and Duncan, 2014). However, few studies have investigated the impact of the golden mean thinking mode of Confucian traditional culture despite it being a good predictor of future behavior. The effect of prosocial motivation on social entrepreneurship orientation is expected to be of different strengths depending on Perceived pressure from external stakeholders.

Under intense pressure from external stakeholders, individuals will constantly monitor the changes in the environment, pay attention to whether their behavior deviates from the goals of stakeholders, adjust their behavior through self-reflection, and take into account various positions and different viewpoints from external stakeholders when solving disputes, adopt a multi-dimensional approach, easy to compromise (Malis et al., 2017). In other words, under the influence of solid pressure perception, individuals should not only be aware of their inner self and adjust their external self-behavior but also change according to the external environment (Hervieux et al., 2012; Shlonsky et al., 2016). Therefore, external stakeholder pressure means that individuals must not only be aware of their inner self but also adjust and be aware of their external self-performance from the perspective of different stakeholders (Dryzin-Amit et al., 2022). Perceived pressure from external stakeholders has rarely been investigated as a moderator in the process of fostering and upgrading social entrepreneurship orientation, even though it is one of the most established and researched variables in entrepreneurship literature (Xiao-Yan, 2013; Parent et al., 2015; Gali et al., 2020).

Based on this logic, this study assumes that the strength of prosocial motivation could be altered by Perceived pressure from external stakeholders (DunCa and Bardacke, 2010; Gvelesiani, 2016). The researchers regard Perceived pressure from external stakeholders as a moderator at the personal level. This view is aligned with social network theory, whereby the influence of social networking on business performance is moderated by cultural factors (Baloglu, 2017; Lückenbach et al., 2019; Hajialiani et al., 2021). Therefore, the researchers predict that there will be a strong relationship between prosocial motivation and social entrepreneurship orientation when Perceived pressure from external stakeholders is high, leading to the following hypothesis:

*Hypothesis5*: Perceived pressure from external stakeholders moderates the relationship between prosocial motivation and social entrepreneurship orientation. This positive relationship is much stronger for those with a high degree of Perceived pressure from external stakeholders.

*H5a*: Perceived pressure from external stakeholders moderates the relationship between prosocial motivation and social entrepreneurship competitive orientation.

*H5b*: Perceived pressure from external stakeholders moderates the relationship between prosocial motivation and social entrepreneurship public-welfare orientation.

Assuming that Perceived pressure from external stakeholders moderates the relationship between prosocial motivation and social entrepreneurship orientation, it is also plausible that an entrepreneur’s characteristics might conditionally affect the strength of the indirect relationship between mindfulness upbringing perception and social entrepreneurship orientation (Wang et al., 2022). In other words, the effect gained from trustworthy networks on social entrepreneurship orientation (mediation effect) may be mediated by Perceived pressure from external stakeholders, thereby demonstrating a moderated mediation effect. As the researchers assume a strong association between prosocial motivation and social entrepreneurship orientation when Perceived pressure from external stakeholders is high, the researchers expect that Perceived pressure from external stakeholders will positively moderate the mediation effect (He et al., 2019). That is, the mediation effect will be stronger when Perceived pressure from external stakeholders is high, as claimed in the following hypothesis:

*Hypothesis6*: Perceived pressure from external stakeholders moderates the indirect effect of mindfulness upbringing perception on social entrepreneurship orientation (via prosocial motivation, respectively). Specifically, prosocial motivation positively mediates the indirect effect when Perceived pressure from external stakeholders is high.

*H6a*: Perceived pressure from external stakeholders moderates the indirect effect of mindfulness upbringing perception on social entrepreneurship competitive orientation (via prosocial motivation, respectively).

*H6b*: Perceived pressure from external stakeholders moderates the indirect effect of mindfulness upbringing perception on social entrepreneurship public-welfare orientation (via prosocial motivation, respectively).

**Figure 1:**
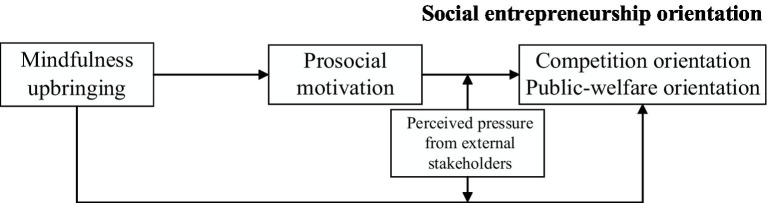
The research model.

Based on the above-proposed hypotheses and the theoretical foundation, the conceptual association among variables is presented below in Figure 1.

## Materials and methods

### Sample selection and data collection

The investigation of this study was divided into two parts: pre-investigation and formal investigation. Before the formal survey, the researchers conducted a pre-survey in February 2021, which targeted 500 social start-ups recommended by China Social Enterprise Forum. These social start-ups are representative enterprises. In February 2021, the researchers took the initiative to contact these enterprises and distributed 490 questionnaires. All items were assessed on a 7-point Likert scale ranging from 1 (strongly disagree) to 7 (strongly agree). The pre-survey was divided into two stages. In the first stage (2021.3–2021.4), researchers interviewed social enterprise founders face-to-face (356) or online (122) and then formed the data of the first round of questionnaire (the first round only included control variables, mindful parenting perception and prosocial motivation). A total of 478 questionnaires were received in the first round. The recovery rate was 97.6%. The second stage of data recovery (August, 2021.8-September, 2021.9) will be carried out about 3 months later, or through a combination of online interview (44) and offline interview (434). The founders of 478 social entrepreneurship enterprises who successfully submitted the questionnaire at the first time point were asked to fill in the questionnaire (the second round of questionnaire only included the perception of mindfulness education, prosocial motivation, social entrepreneurship motivation, and external pressure perception), and 476 questionnaires were recovered, with a recovery rate of 99.6%. Since the questionnaires administered at both stages included both mindful parenting and prosocial motivation, the researchers compared the responses to the questionnaires received at the two stages for the same social start-up (each social start-up has a unique ID number). The questionnaire with the same score of these two variables in the two rounds of answers was retained as one valid questionnaire, and a total of 380 valid questionnaires were collected during the pre-survey.

The questionnaire collected from the pre-survey was found to be of good quality after inspection. Therefore, this means that the scale of variables has good external validity, is in line with the Chinese situation, and is reasonable. We can conduct a formal investigation, which was conducted from the end of September 2021 to February 2022.

Since 2015, the research team has focused on social entrepreneurship and established cooperative relationships with domestic recognized social entrepreneurship research and service institutions such as EN-pai Public Welfare Platform and China Social Enterprise Forum (Annual Conference), accumulating rich case data. These institutions and forums provide communication and service platforms for social entrepreneurship participants across the country, giving great support to the random selection of research objects in this study. The object of the formal survey was 1,000 social start-ups randomly selected from the database of the national recognized social entrepreneurship research and service institutions such as Enpai Public Welfare Platform and China Social Enterprise Forum (Annual Meeting).

To clarify the causal inference and alleviate the problem of standard method variance (CMV), the researchers separated measurement occasions (Widyalankara, 2016). The data were collected at three time points, one month apart. The formal questionnaire was completed in three stages (T1/T2/T3). In order to avoid the deviation of social desirability, the data in the three stages were emphasized to be used only for research and kept strictly confidential.

The first round (T1) survey measured self-reported mindfulness upbringing perception (the level of mindfulness upbringing perception from their parents during childhood and adolescence received) and collected demographic information of the participants. At this stage, 1,000 questionnaires were distributed online (138) and offline (862), and 890 questionnaires were collected (136 online and 754 offline), with an effective recovery rate of 89%.

The second round (T2) survey measured prosocial motivation and perceived pressure from external stakeholders. The questionnaire at this stage is for social enterprise founders who successfully submit the questionnaire at the first point in time to fill out. A total of 890 questionnaires were distributed through online distribution (134) and offline distribution (756), and a total of 792 questionnaires were recovered (101 online and 691 offline), and the effective recovery rate was 88.9%.

The third round (T3) survey measured social entrepreneurship orientation. No monetary incentive was offered to the participants. The questionnaire at this stage is for social enterprise founders who successfully submit the questionnaire at the second time point to fill out. At this stage, a total of 792 questionnaires were distributed through online distribution (99) and offline distribution (693), and a total of 690 questionnaires were recovered (80 online and 610 offline). The effective recovery rate was 87.1%. After excluding missing data and outliers based on boxplot analyses, 558 responses were analyzed.

**Figure 2:**
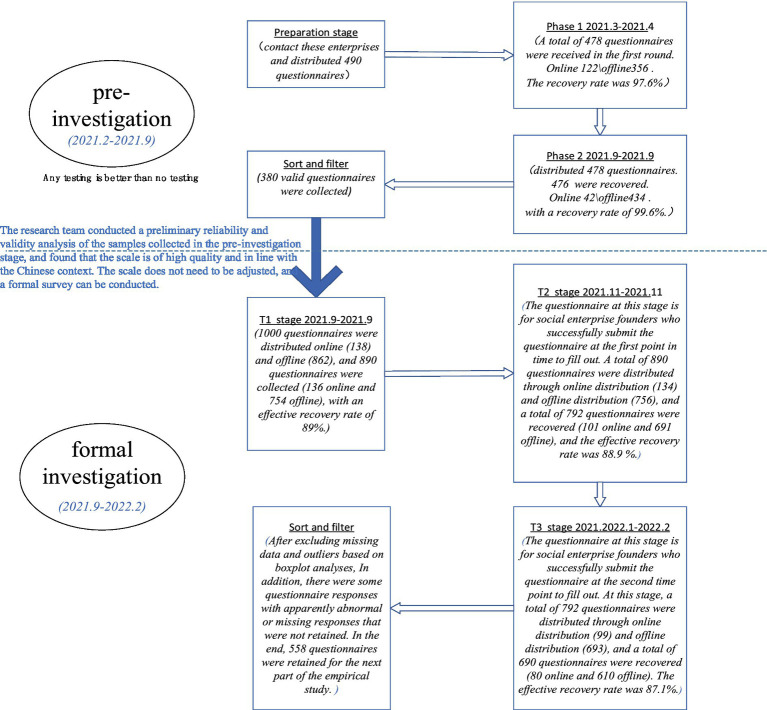
The detailed process of data collection.

The detailed process of data collection and the number of questionnaires collected at each time point are shown in the Figure 2.

Among these participants, the majority were male (50.7%). The researchers calculated the following statistics based on demographic data. The results of the descriptive statistical analysis of sample enterprises are shown in Table 1. 240 respondents were in their 30s (43.0%), 218 respondents in their 40s (39.1%), 67 respondents in their 50s (12.0%), and 33 respondents were in their 60s (5.9%). Among all respondents, 196 had a bachelor’s degree or higher (35.1%). “Electric, electronics, communication and precision “was the most popular industrial category, accounting for 47.7% of all respondents. Regarding their work experience, 40.9% of all respondents had 1–3 years of experience working within the same industry. Table 1 presents the demographic information of the research sample.

**Table 1:** Demographics of survey respondents (N = 558).

Variable	Category	N	Percentage (%)
gender	Male	283	50.7%
	Female	275	49.3%
Age	The 20s	0	0.0%
	The 30s	240	43.0%
	The 40s	218	39.1%
	The 50s	67	12.0%
	The 60s	33	5.9%
Education	Junior college and below	20	3.6%
	Bachelor’s degree	196	35.1%
	Master’s degree	235	42.1%
	Doctoral degree	107	19.2%
Industry type	Nonmetal, metals, machine equipment	163	29.2%
	Computer and office machine	87	15.6%
	Electric, electronics, communication and precision	266	47.7%
	Daily supplies	35	6.3%
	Other	7	1.3%
Work experience in the same industry	1–3	228	40.9%
	4–5	160	28.7%
	6–8	139	24.9%
	9-	31	5.6%

Through the F test and T test of the online and offline overall sample data, it is found that the *p* values are all greater than the significance level of 0.05, indicating that there is no significant difference in the data, and the mixed use will not have a great impact on the reliability of the research results.

### Variable measurement

The perception of mindfulness upbringing refers to the respondents’ perception of the extent to which their parents practiced mindfulness upbringing during their childhood family education (Mansehra, 2018). Parents’ mindful upbringing plays an essential role in developing their children’s mental health and social adaptability (Duarte et al., 2019). The measurement of entrepreneurs’ mindfulness upbringing perception was based on the rationale proposed by (Rodrigo et al., 2021). Upbringing perception mainly includes five dimensions: attentive listening of parents, non-judgmental acceptance of themselves, and their children, emotional awareness of themselves and their children, self-regulation in parent–child relationship, and compassion for themselves and their children. Accordingly, the measure of mindful upbringing perception is divided into five dimensions. Items included “In the family, you can feel that your parents are listening attentively to what you express”; “your parents are more accepting of themselves and you without judgment”; “your parents are more aware of their emotions and your emotions”; “you can feel parents’ self-regulation in parent–child relationship”; “you can feel parents’ compassion for themselves and you.” For this construct, Cronbach’s Alpha was 0.990. According to Chandra (2016), reliability of 0.70 or better is recommended (Chandra, 2016). Hence, this value has sound scale reliability.

In essence, prosocial behavior belongs to a broad category of interpersonal interaction (Rodrigo et al., 2021), including voluntary activities of helping others, sharing and cooperating to safeguard others’ interests (Crick et al., 2010). Prosocial behavior is characterized by social interaction (Honig and Hopp, 2019), high social approval (Crick et al., 2010) altruism, and reciprocity (Fatima and Bilal, 2019). The prosocial motivation scale developed by Crick et al. (2010) was used to measure the degree of the willingness of social entrepreneurs to make efforts to meet their interests from the perspective of the interests of the public. The prosocial motivation scale including five items, sample items included “I want to make a positive impact on others through my work,” “I’m willing to volunteer my time and energy, not to get paid more.” For this construct, Cronbach’s alpha was 0.987.

Perceived pressure from external stakeholder’s scale was developed by Garcés-Ayerbe et al. (2012), which measures external stakeholder pressure closely related to the operational activities of social enterprises from four aspects: customers, competitors, partners, and government, included four items. Sample items were including “Our customers prefer products and services with social impact/social value,” “Competitors’ products or services receive positive social evaluations compared to ours,” “Our partners pay great attention to social impact and solve social problems in their products and services,” “The local government prefers social enterprises with positive social impact and provides certain policy support.” For this construct, Cronbach’s alpha was 0.973.

Social entrepreneurship orientation is measured from two dimensions: competition orientation and public welfare orientation. ①The competitive orientation scale is developed by Narver and Slater (2017). Competition orientation reflects the degree of economic emphasis in the process of social entrepreneurship, a 7-point Likert scale with three items. Sample items included “I want my company to maintain a sense of superiority in the industry, become the center of attention, and continue to be seen and noticed,” “When I run a business, I tend to turn work into a competition,” “I hope my company can beat other companies in the industry and become a winner.” For this construct, Cronbach’s alpha was 0.985. ②The public-welfare orientation scale adopts Cooke’s organizational culture scale (Cooke and Rousseau, 1988) and other scales of humanitarian care (Darmanto and Bukirom, 2021), as well as the public-welfare orientation scale revised according to the survey. The public-welfare orientation reflects the emphasis of the social entrepreneurship process, which is a 7-point Likert scale containing five items. Sample items included “My business tries to help others grow and develop,” “I hope my company can beat other companies in the industry and become a winner,” “I hope my company can solve social conflicts constructively,” “My business can recognize and care for the needs of others in its operation.” For this construct, Cronbach’s Alpha was 0.991.

Control variables. The control variables in this study include the industry field of social entrepreneurs, Work experience in the same industry, and the gender, age and education level of social entrepreneurs (Cooke and Rousseau, 1988; Garcés-Ayerbe et al., 2012; Narver and Slater, 2017; Parajuli et al., 2019; Halberstadt et al., 2020).

## Empirical analysis and results

To test the hypotheses this study has used a moderated mediation model. It is a statistical method that comprises mathematical and statistical approaches for examining data to identify relationships between variables (Duarte et al., 2019; Moisés et al., 2019). SPSS and PROCESS3.3 were used to analyze the data in this study. These are useful for measuring mediating and moderating effects and are suitable for the exploratory nature of study analysis (Korneiko, 2017). In recent years the number of published articles using a moderated mediation model increased significantly.

### Reliability analysis

It is essential to check the reliability and validity of measurement tools (de Waal and Suchak, 2010). Reliability analysis verifies the internal consistency of the scale, that is, whether different items can measure the same content or concept independently. Cronbach’s Alpha coefficient is mainly used in this study to investigate the internal consistency of the scale. Cronbach’s Alpha coefficient is between 0 and 1. If the α coefficient does not exceed 0.6, internal reliability is generally considered inadequate. When the α coefficient reaches 0.7–0.8, the scale has considerable reliability. When the α coefficient comes 0.8–0.9, the scale’s reliability is excellent (Alexander, 2017). As shown in Table 2, the total reliability of this study is 0.986, greater than 0.9. Cronbach’s Alpha of all dimensions of the scale is greater than 0.9. The results show that the scale and dimensions have high reliability, good stability and consistency and can be used for in-depth analysis means detailed analysis of mediating and moderating effects between variables in section 3.4 and 3.5.

**Table 2:** The specific content and reliability test of each dimension of the scale (N = 558).

Variable		Items	CITC	Cronbach’s Alpha	
X Mindfulness upbringing perception	5	In the family, you can feel that your parents are listening attentively to what you express	0.833	0.984	0.990
		Your parents are more accepting of themselves and you without judgment	0.849	0.984	
		Your parents are more aware of their emotions and your emotions	0.832	0.984	
		You can feel parents’ self-regulation in the parent–child relationship	0.821	0.984	
		You can feel parents’ compassion for themselves, and you	0.841	0.984	
M Prosocial motivation	5	I want to make a positive impact on others through my work	0.797	0.984	0.987
		I’m willing to volunteer my time and energy, not to get paid more	0.790	0.984	
		I do not help people with the goal to receive their thanks and return	0.772	0.984	
		I tend to help others, even if there is no benefit	0.782	0.984	
		I think it’s best to help people when they do not know	0.815	0.984	
W Perceived pressure from external stakeholders	4	Our customers prefer products and services with social impact/social value	0.709	0.984	0.973
		Competitors’ products or services receive positive social evaluations compared to ours	0.689	0.984	
		Our partners pay great attention to social impact and solve social problems in their products and services	0.687	0.984	
		The local government prefers social enterprises with positive social impact and provides particular policy support	0.688	0.984	
Y1 Social entrepreneurship competition orientation	3	I want my company to maintain a sense of superiority in the industry, become the center of attention, and continue to be seen and noticed	0.843	0.984	0.985
		When I run a business, I tend to turn work into a competition	0.856	0.983	
		I hope my company can beat other companies in the industry and become a winner	0.848	0.984	
		Our business strategy orientation is driven by our belief in how to create more significant value for our customers	0.827	0.984	
Y2 Social entrepreneurship public-welfare orientation	5	My business tries to help others grow and develop	0.881	0.983	0.991
		I want my business to give positive rewards to others	0.878	0.983	
		I hope my company can solve social conflicts constructively	0.885	0.983	
		My business can recognize and care for the needs of others in its operation	0.891	0.983	

*N = 558*; independent variable X stands for mindfulness upbringing perception; dependent variable Y1 stands for social entrepreneurship competition orientation; dependent variable Y2 stands for social entrepreneurship public-welfare orientation; mediator variable M stands for prosocial motivation; moderator variable W stands for perceived pressure from external stakeholders.

### Validity analysis

KMO sample measure and Bartlett sphere test should be used to verify partial correlation and simple correlation coefficient of various variable items before factor analysis (Wiguna and Manzilati, 2014). Data are suitable for factor analysis only when correlation is high (Dwivedi and Weerawardena, 2018). KMO and Bartlett test results of all variables in this study are shown in Appendix 1. KMO value is 0.938, greater than 0.9, indicating that the data are suitable for factor analysis (Sulphey and Salim, 2020). Bartlett sphericity test chi-square value is 25705.260 (*p* < 0.01), indicating that the relationship between items of user variables is good and factor analysis can be carried out. Explain the eigenvalues of the total variance observation scale and the sum of the squares of the rotating loads as well as the cumulative percentage of the main observation items. More than 50% indicates compliance with factor analysis requirements (Fatima and Bilal, 2019). As can be seen from the variance interpretation rate after rotation (Appendix 1), a total of five factors were extracted, accounting for 94.497% of the total variance, more than 50%, indicating that the extracted 5 factors could better explain the information contained in the original variable. From the factor load result, the factor load of each dimension item was greater than 0.6, and each item was within its original defined dimension without variable confusion, indicating that the model had high structural validity (Brndle et al., 2019). Then, confirmative factor analysis questionnaire structure validity was used in AMOS24.0. The model fitting results showed that the absolute fit index was demonstrated in Appendix 1, with GFI, AGFI, NFI, IFI, TLI, and CFI all greater than 0.8, indicating that the structure validity passed the test.

### Correlation analysis

Table 2 shows the descriptive statistics and correlations for the variables included in the study. In the correlation analysis of various numerical variables, the commonly used statistical analysis method is the Pearson correlation coefficient (Fina et al., 2017). Academics use it to measure correlations between economic phenomenon or variables. The academia reveals and reflects the correlation between different things or variables through numerical quantification (Garon and Nassif, 2021). As seen from Table 3, the mean values of mindfulness upbringing perception, prosocial motivation, social entrepreneurship competition orientation, and social entrepreneurship public-welfare orientation are 4.05, 3.62, 4.06, and 4.99, respectively. These values are in the middle. It indicates that the mindfulness upbringing perception, prosocial motivation, social entrepreneurship competition orientation and social entrepreneurship public-welfare orientation need to be improved. The average level of perceived pressure from external stakeholders is 2.88, indicating that perceived pressure from external stakeholders is at a low level. All the variables showed a positive correlation. As shown in Table 3. This provides preliminary support for the above research hypothesis1,2,3,4.

**Table 3:** Correlation test (N = 558).

	Mean	SD	Mindfulness upbringing perception	Prosocial motivation	Perceived pressure from external stakeholders	Competition orientation	Public-welfare orientation
Mindfulness upbringing perception	4.05	1.940	1				
Prosocial motivation	3.62	1.742	0.693^**^	1			
Perceived pressure from external stakeholders	2.88	1.493	0.556^**^	0.391^**^	1		
Competition orientation	4.06	1.608	0.712^**^	0.689^**^	0.684^**^	1	
Public-welfare orientation	4.99	1.740	0.722^**^	0.725^**^	0.722^**^	0.749^**^	1

* Correlation is significant at *p* < 0.05 (two-tailed test).

** Correlation is significant at *p* < 0.01 (two-tailed test).

### Mediation effect of prosocial motivation

The researchers adopted Bednall et al. (2013) suggestion to test the mediation effect of prosocial motivation between mindfulness upbringing perception and social entrepreneurship orientation (Social entrepreneurship competition orientation& public-welfare orientation). According to Bednall et al., four requirements need to be met to assess the mediation effect. First, the independent variable X and the mediation variable M should each be regressed on the dependent variable Y (Y1&Y2). The variable X should also be regressed on the variable M. Partial mediation impact is confirmed if the variable X remains powerful and its effect becomes smaller while controlling the variable M. Full mediation effect occurs if the variable X is no longer significant (Details et al., 2019).

Hierarchical regression was used to test the direct effects of mindfulness upbringing perception on competitive orientation and public welfare exposure. The results are shown in Table 3. According to model 2, mindfulness upbringing perception has a substantial positive effect on competitive orientation (β = 0.590, *p* < 0.001), and R^2 in model 2 is significantly increased compared with R^2 in model 1, and the change of R^2 is significant at 0.01 level, indicating that mindfulness upbringing perception has a significant effect on competitive orientation compared with control variables. Hypothesis 1A is verified. Similarly, it can be seen from Model 6 that mindfulness upbringing perception has a significant positive effect on public-welfare orientation (β = 0.647, *p* < 0.001), and the R^2 of Model 6 is significantly higher than that of model 5, and the change of R^2 is significant at the level of 0.01, indicating that compared with the control variable, mindfulness upbringing perception has a significant impact on public-welfare orientation. Hypothesis 1b is verified. In addition, the age of social entrepreneurs has a positive effect on the competitiveness orientation, which also confirms that older entrepreneurs are more conducive to the competitiveness orientation, which is also consistent with the phenomenon that a large percentage of successful social entrepreneurs are middle-aged. Still, age has no significant positive effect on the improvement of public-welfare exposure.

Then the researchers examine the mediating impact of prosocial motivation on mindfulness upbringing perception and competitive orientation. According to Model 2 in Table 4, mindfulness upbringing perception has a significant positive impact on competitive orientation (β = 0.590, *p* < 0.001). According to model 3 in Table 3, mindfulness upbringing perception has a significant positive impact on prosocial motivation (β = 0.6257, *p* < 0.001). The results show that prosocial motivation has a substantial positive effect on competitive orientation (β = 0.3436, *p* < 0.001). Still, the effect of mindfulness upbringing perception on competitive orientation is still significant, but the regression coefficient is 0.590 (β = 0.590, *p* < 0.001) decreased to 0.3748 (β = 0.3748, *p* < 0.001), suggesting that prosocial motivation plays a partial mediating role in the relationship between mindfulness upbringing perception and competitive orientation. Similarly, according to Models 7 and 8, prosocial motivation plays a partially mediating role in the relationship between mindfulness upbringing perception and public welfare orientation.

**Table 4:** Test results of direct effect and mediating effect (N = 558).

Competition orientation					Public-welfare orientation			
	M1	M2	M3	M4	M5	M6	M7	M8
	Control Variables→Competition orientation	Control Variables+Mindfulness upbringing perception → competitive orientation	Control Variables+Mindfulness upbringing perception→Prosocial motivation	Control Variables+Mindfulness upbringing perception+Prosocial motivation→Competition orientation	Control Variables→Public-welfare orientation	Control Variables+Mindfulness upbringing perception →public-welfare orientation	Control Variables+Mindfulness upbringing perception→Prosocial motivation	Control Variables+Mindfulness upbringing perception+Prosocial motivation→Public-welfare orientation
Constant	3.852^**^(7.333)	1.440^*^ (3.710)	1.5913^**^ (3.6889)	0.8927^*^ (2.4421)	5.610 (9.883)	2.963^**^(7.183)	1.5913^**^ (3.6889)	2.2871^**^(6.0705)
Entrepreneurial experience	0.109 (0.659)	−0.030 −0.261	−0.2262 (−1.7491)	0.0474 (0.4387)	0.067 (0.374)	−0.08 (−0.696)	−0.2262 (1.7491)	0.0101(0.0905)
Age	0.184^*^(1.857)	0.198^*^ (2.845)	0.0860(1.1130)	0.1681^**^ (2.6101)	0.130(1.211)	0.145(1.957)	0.0860 (1.1130)	0.1081(1.6281)
Education Level	−0.016 (0.184)	0.021 0.341	0.0700 (1.0152)	−0.0029 (−0.0503)	−0.087 (−0.911)	−0.046 (−0.698)	0.0700 (1.0152)	−0.0758 (1.2788)
Regions for Entrepreneurship	0.034(0.192)	−0.003 −0.026	−0.1476 (−1.0713)	0.0475 (0.4135)	−0.213 (−1.115)	−0.254 (−1.925)	−0.1476 (−1.071)	−0.1909 (1.6131)
Enterprise scale	−0.146 (1.683)	−0.079 −1.299	−0.0935 (−1.3816)	−0.0470 (−0.8316)	−0.177 (−1.892)	−0.104 (−1.606)	−0.0935 (−1.382)	−0.0642 (1.1034)
Mindfulness upbringing perception		0.590^*^ (19.707)	0.6257^**^ (18.8053)	0.3748^**^ (9.6951)		0.647^**^(20.333)	0.6257^**^ (18.805)	0.3811^**^(9.5661)
Prosocial motivation				0.3436^**^ (7.9739)				0.4249^**^(9.5648)
$R^{2}$	0.016		0.4925	0.5884	0.019	0.523	0.4925	0.6265
Adjusted$R^{2}$	0.003		0.476	0.584	0.006	0.514	0.476	0.613
F·	1.239，*p* = 0.000	60.317, *p* = 0.000	60.3207, *p* = 0.000	75.9754, *p* = 0.000	1.442, *p* = 0.000	61.330, *p* = 0.000	60.3207, *p* = 0.000	89.1480, *p* = 0.000

*N = 558*; independent variable X stands for mindfulness upbringing perception; dependent variable Y1 stands for social entrepreneurship competition orientation; dependent variable Y2 stands for social entrepreneurship public-welfare orientation; mediator variable M stands for prosocial motivation; moderator variable W stands for perceived pressure from external stakeholders.

* Correlation is significant at p < 0.05 (two-tailed test).

** Correlation is significant at p < 0.01 (two-tailed test).

Moreover, the Bootstrap method was then used to examine further the mediating effect of the model (Sabbagh, 2018). The Bootstrap aping was observed 1,000 times. As table 5 showed, the 95% confidence interval CI = (0.1559, 0.2774), excluding 0, showed that the indirect impact of mindfulness upbringing perception on competitive exposure was substantial through prosocial motivation. The effect value was 0.2150, and the direct effect of mindfulness upbringing perception in the relationship between competitive orientation was significant (95% confidence interval CI = 0.2988, 0.4508), excluding 0, the effect value was 0.3748, indicating that the partial mediating effect of prosocial motivation in the relationship between mindfulness upbringing perception and competitive orientation was supported again. Similarly, the partial mediating role of prosocial motivation in the relationship between mindfulness upbringing perception and public welfare orientation was supported again. The results of mediating effect further indicated that prosocial motivation had a partial mediating effect between mindfulness upbringing perception and competitive orientation & public welfare orientation.

**Table 5:** Mediating effect Bootstrapping test results (N = 558).

Effect of path	Coefficient	Coefficient	Boot 95% CI
Mindfulness upbringing perception → prosocial motivation → competitive orientation	Direct effect	0.3748	(0.2988， 0.4508)
	Indirect effect	0.2150	(0.1559， 0.2774)
Mindfulness upbringing perception → prosocial motivation → public-welfare orientation	Direct effect	0.3811	(0.3028， 0.4595)
	Indirect effect	0.2659	(0.2078， 0.3289)

### Moderating effect of perceived pressure from external stakeholders

As shown in Table 6, M9 and M10 take competition orientation and public welfare orientation as dependent variables, respectively. Based on M3 and M7, perceived pressure from external stakeholders and the intersection term (perceived pressure from external stakeholders & mindfulness upbringing perception) are added. As can be seen from Table M9 and M10, the regression coefficient of the intersection term (perceived pressure from external stakeholders & mindfulness upbringing perception) on competition orientation and public welfare orientation both have reached significant levels. Therefore, perceived pressure from external stakeholders negatively moderates the relationship between mindfulness parenting and social entrepreneurship orientation (competition orientation & public-welfare orientation).

**Table 6:** Test of moderating effect of the main effect (N = 558).

Dependent variable	Competition orientation	Public-welfare orientation
	M9	M10
Constant	0.4961(1.2197)	0.9596^*^(2.4375)
Entrepreneurial experience	−0.0850(−0.8354)	−0.1208(−1.2261)
Age	0.1147(1.8793)	0.0442(0.7485)
Education Level	0.0298(0.5510)	−0.0339(−0.6478)
Regions for Entrepreneurship	0.0945(0.8725)	−0.1491 (−1.4218)
Enterprise scale	−0.0501(−0.9416)	−0.0769(−1.4953)
Mindfulness upbringing perception	0.5103^**^(8.0701)	0.7675^**^ (12.5383)
Perceived pressure from external stakeholders	0.6316^**^(6.1876)	1.1254^**^(11.3900)
Mindfulness upbringing perception* perceived pressure from external stakeholders	−0.0408^**^(−1.9960)	−0.1292^**^ (−6.5379)
$R^{2}$	0.6364	0.7090
F	81.1733, *p* = 0.000	113.0019, *p* = 0.000

** *p* < 0.01; *p < 0.05.

**Figure 3:**
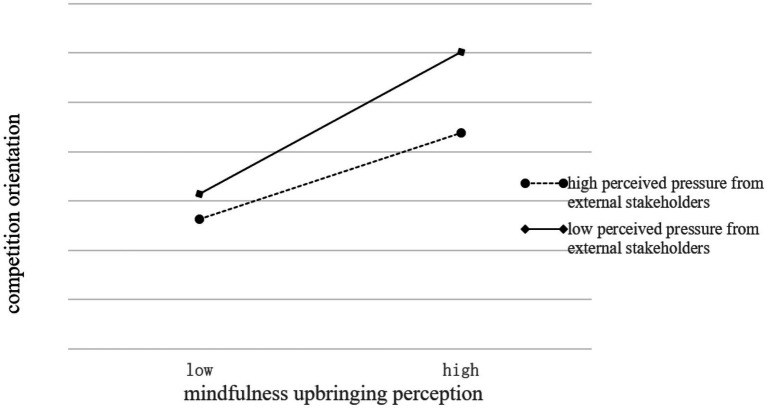
The moderating effect chart (mindfulness upbringing perception and competition orientation).

**Figure 4:**
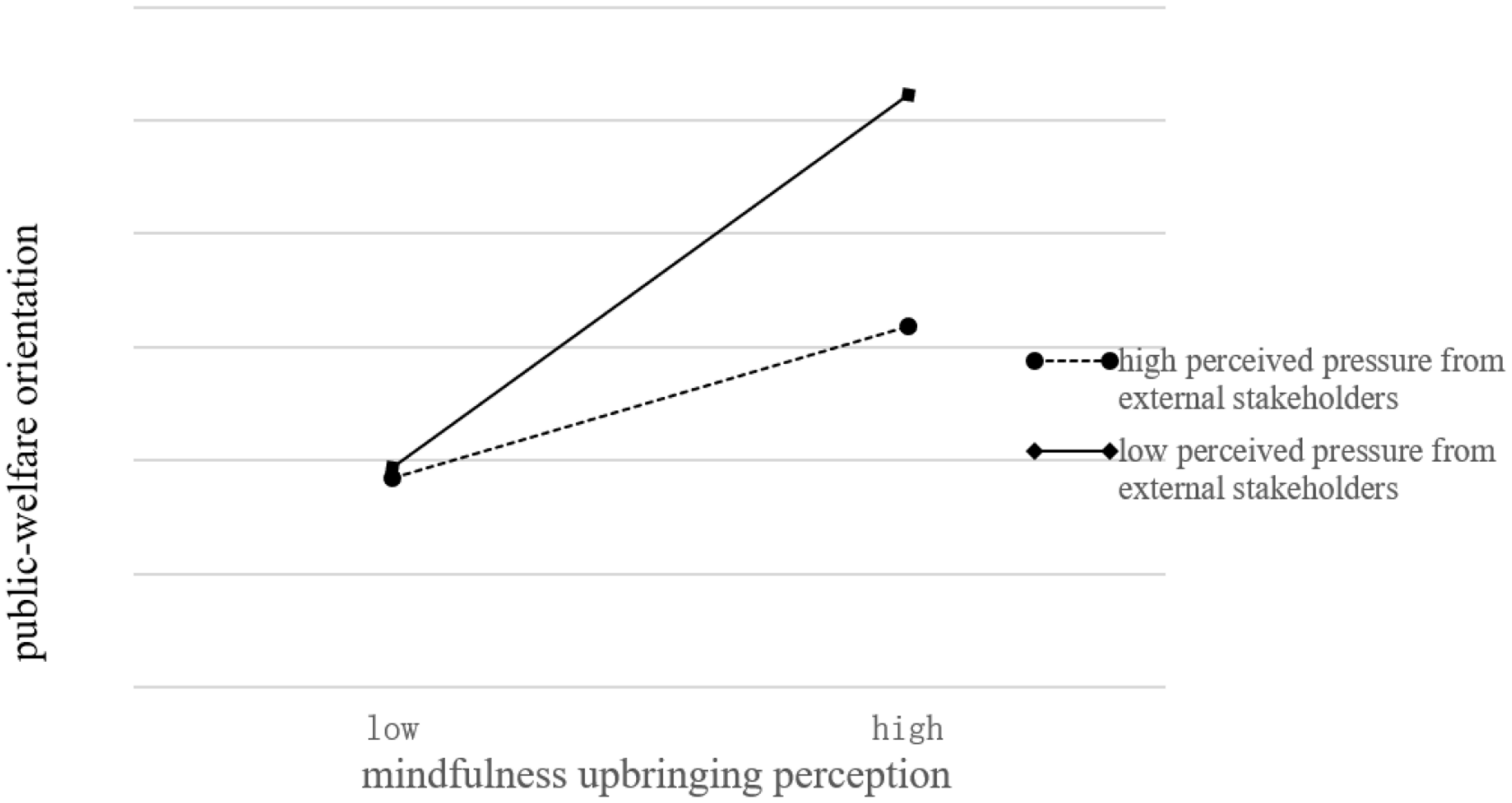
The moderating effect chart (mindfulness upbringing perception and public-welfare orientation).

To show the moderating effect of perceived pressure from external stakeholders more clearly, the moderating effect chart was drawn. To more clearly show the moderating effect in perceived pressure from external stakeholders on the relationship between mindfulness upbringing perception and social entrepreneurship orientation (competition orientation and public-welfare orientation), the researchers describe the difference of the moderating effect of mindfulness upbringing perception on social entrepreneurship orientation in different levels of perceived pressure from external stakeholders with one standard deviation higher and one standard deviation lower than the mean, respectively. As shown in Figures 3, 4, compared with social entrepreneurs with a higher level of perceived pressure from external stakeholders, the regression line of competition orientation and public welfare orientation of social entrepreneurs with a lower level of perceived pressure from external stakeholders presents steeper trends. In lower levels of perceived pressure from external stakeholders, the positive effect of mindfulness upbringing perception on social entrepreneurship orientation is more substantial; However, at a higher level of perceived pressure from external stakeholders, the impact of mindfulness upbringing perception on social entrepreneurship orientation has little difference. In conclusion, perceived pressure from external stakeholders inhibits the positive effect of mindfulness upbringing perception on social entrepreneurship orientation (competition orientation and public-welfare orientation).

To further verify the significance of the above moderating effect, a simple slope test and slope difference test are conducted, and the results are shown in Table 7. When social entrepreneurs were under a higher level of perceived pressure from external stakeholders, the positive effect of mindfulness upbringing perception on competitive orientation was lower (β = 0.3167, *p* < 0.001), and when social entrepreneurs were under lower-level perceived pressure from external stakeholders, the positive effect of mindfulness upbringing perception on competitive orientation was significantly increased (β = 0.4594, *p* < 0.001). When social entrepreneurs are under different levels of perceived pressure from external stakeholders, the effect of mindful cultivation on competitive orientation is significantly different. Similarly, when social entrepreneurs are under a higher and lower-level perceived pressure from external stakeholders, there are significant differences in the impact of mindfulness upbringing perception on public-service orientation. Therefore, perceived pressure from external stakeholders has a negative moderating effect on the main result.

**Table 7:** Simple slope test and slope difference test results (N = 558).

Effect of path	Moderator	Coefficient	S.E	LLCI-ULCI(95% confidence interval)
Mindfulness upbringing perception → competitive orientation	High level of perceived pressure from external stakeholders	0.3167	0.0525	(0.2135, 0.4200)
	Mean level of perceived pressure from external stakeholders	0.3881	0.0320	(0.3251, 0.4510)
	Low level of perceived pressure from external stakeholders	0.4594	0.0430	(0.3748, 0.5439)
Mindfulness upbringing perception →public-welfare orientation	High level of perceived pressure from external stakeholders	0.1537	0.0508	(0.0538， 0.2537)
	Mean level of perceived pressure from external stakeholders	0.3799	0.0310	(0.3189， 0.4408)
	Low level of perceived pressure from external stakeholders	0.6060	0.0416	(0.5241， 0.6878)

To further verify the moderated mediating effects. In this study, the PROCESS plug-in was used to test the mediated role according to Wen Zhonglin et al. The researchers adopted the Bootstrapping method to test the significance of the mediating effect of perceived pressure from external stakeholders at different levels. The effective value of the moderating effect was obtained. The results are shown in Table 8. When social entrepreneurs are under high-level pressure from external stakeholders, the indirect impact of mindfulness upbringing perception on competitive orientation and public welfare orientation is significant through prosocial motivation, 95% confidence interval is CI = (0.1420, 0.2903), CI = (0.0910, 0.1930), excluding 0. The effect values were 0.2124 and 0.1390. With the change of perceived pressure from external stakeholder’s level from high to low, the indirect effect of mindfulness upbringing perception on competitive orientation and public benefit orientation increased from 0.2117 to 0.2124 and 0.1390 to 0.3684, respectively, with a 95% confidence interval CI = (0. 1,377, 0. 0. 2,873), CI = (0.2892, 0.4451), excluding 0. Therefore, perceived pressure from external stakeholders significantly negatively moderates the mediating effect of prosocial motivation in the relationship between mindfulness parenting and social entrepreneurship orientation (competition orientation & public welfare orientation).

**Table 8:** The moderated mediation test sheet (N = 558).

The indirect effect	Moderator	Coefficient	S.E	LLCI-ULCI(95% confidence interval)
Mindfulness upbringing perception → prosocial motivation → competitive orientation	High level of perceived pressure from external stakeholders(M + 1SD)	0.2117	0 0.0378	(0 0.1420， 0.2903)
	Mean level of perceived pressure from external stakeholders(M)	0.2121	0 0.0267	(0.1595， 0.2667)
	Low level of perceived pressure from external stakeholders(M-1SD)	0.2124	0.0387	(0 0.1377, 0 0.2873)
Mindfulness upbringing perception → prosocial motivation → public-welfare orientation	High level of perceived pressure from external stakeholders(M + 1SD)	0.1390	0 0.0256	(0.0910, 0.1930)
	Mean level of perceived pressure from external stakeholders(M)	0.2537	0.0229	(0.2097, 0.2985)
	Low level of perceived pressure from external stakeholders(M-1SD)	0.3684	0.0401	(0.2892， 0.4451)

## Discussion

### Implications

#### Theoretical implications

The possible theoretical contributions of this study are mainly reflected in three aspects:

First, it deepens the research on the connotation, impact and effect mechanism of mindfulness upbringing perception. The academic research has not reached a consensus on the connotation definition and functional characteristics of social entrepreneurs’ mindfulness upbringing perception in the Context of Chinese culture, and there are debates on capital, culture, and ethics. Different from previous research, this article follows the evolution history and injection time development characteristics based on social learning theory thoroughly, discusses mindfulness breeding perception of social entrepreneurs to social entrepreneurship orientation (competition orientation and public interest orientation) and the internal mechanism, the influence of this for mindfulness breeding nature has specific theoretical meaning. It also enriches the relevant researches on the mechanism and consequences of social entrepreneurs’ perception of family mindfulness upbringing.

Second, it has enriched the research on antecedent variables of social entrepreneurship orientation (competitive orientation and public welfare orientation) from the perspective of individual factors. It is of great significance to explore the antecedent variables of social entrepreneurship orientation (competitive orientation and public welfare orientation) to deeply understand the origin of social entrepreneurship orientation (competitive orientation and public-welfare orientation) and to cultivate social entrepreneurship orientation (competitive orientation and public welfare orientation). Existing researches mainly focus on the influence of individual spiritual traits or personal value such as self-confidence, optimism, and hope, and the resulting psychological resources such as trust and commitment on forming social entrepreneurship orientation (competitive orientation and public-welfare orientation). This study further discusses the influence and internal mechanism of higher individual values, such as the prosocial motivation of social entrepreneurs. And this study which forms an essential supplement to existing relevant research.

Thirdly, it finds the “key” connecting the prosocial motivation of social entrepreneurs with the orientation of social entrepreneurship (competition orientation and public-welfare orientation). In this study, the prosocial motivation of social entrepreneurs is defined as a kind of introspective, result-oriented, and future-oriented. It is manifested in the specific entrepreneurial values that inspire and integrate their stakeholders to jointly create social value and meet the social needs that cannot be completed by the existing system, market, and government. Most current studies on the relationship between individual values and entrepreneurial orientation explore the internal relationship between them from the perspective of social capital accumulation. This study, referring to the literature of motivational information processing theory and emotional contagion theory, discusses the mediating effect of prosocial motivation on the relationship between social entrepreneurs’ mindfulness upbringing perception and social entrepreneurship orientation (competitive orientation and public-welfare orientation). This study also builds a new bridge for the study of the relationship between individual values and entrepreneurship orientation.

#### Practical implications

In practice, the results of this study have implications for social entrepreneurs to objectively examine their own internal and external conditions, improve the decision-making level of social entrepreneurship, and strengthen the guarantee conditions of social entrepreneurship orientation.

1. Pay attention to the role of mindfulness cultivation perception and attach importance to prosocial motivation. Children’s perception of their parents’ mindful upbringing plays a vital role in developing their prosocial motivation and social adaptability. First, parents should improve their awareness and management of their feelings. Parents should reduce negative emotions or out-of-control emotions caused by children’s destructive behaviors or attitudes in the process of raising children, adjust themselves in time, and avoid negative feelings or ignore them when children express negative emotions. Parents’ conscious choice of appropriate ways to respond to their children is conducive to forming children’s pro-social motivation. It can also strengthen their social entrepreneurship public welfare motivation and competitive motivation. Secondly, parents should not be sensitive to the content of their children’s speech, but should also effectively use nonverbal cues to improve their understanding and sensitivity to their children’s emotional expression and understanding by judging their children’s voice tone, facial expressions, and body language. Parents should not only convey understanding and acceptance to their children, but also provide clear codes of conduct and discipline rules for their children, and set expectations for their children. Finally, mindfulness training or curriculum programs improve the level of mindfulness, promote the formation of positive parent–child interaction, promote pro-social motivation, social entrepreneurship, public-welfare motivation, and competitive motivation.

2. Be alert to the intensity of stakeholder pressure on social entrepreneurs. To treat the environmental pressure from stakeholders, social entrepreneurs are encouraged to develop stakeholder-centered policies and corporate strategies, emphasizing communication, to mitigate the negative effect of stress on public-welfare orientation and competition orientation.

### Limitations and future research directions

Inevitably, there are some limitations in this study. First, in terms of sample data, this study collects the data needed for the research through a sample survey of Chinese social entrepreneurship enterprises. However, due to the inherent defects of the sampling survey and the impact of the epidemic, the effective recovery rate in some regions is low, leading to some deviation between the statistical distribution of sample enterprises and the actual situation, which has a particular impact on the representativeness of sample data and may reduce the universality of the research conclusions. In the future, the sampling will be more scientific, and the survey scope will be expanded. Longitudinal multi-point tracking research will be used to more accurately and deeply understand the relationship between mindfulness parenting perception and social entrepreneurship orientation. Second, this study only focuses on prosocial motivation as the “key” to opening the black box of the relationship between mindfulness upbringing perception and social entrepreneurship orientation and fails to comprehensively and systematically reveal the complex mechanism of the relationship between mindfulness upbringing perceptions and social entrepreneurship orientation. There are more transfer factors and complex mechanisms between the perception of mindfulness upbringing and different orientation of social entrepreneurship at the individual level. Future research needs to look for new “keys” from different perspectives.

## Conclusion

The purpose of this study is to explore the internal mechanism of the impact of mindfulness parenting perception on social entrepreneur orientation. To reveal the internal mechanism, the researchers propose a moderated and mediation model of prosocial motivation and perceived pressure from external stakeholders. Using survey data from social entrepreneurs in China, hierarchical regression analysis and bootstrapping model are adapted to test and verify mediation and moderation effects. (1) The results show that mindfulness upbringing perception indeed positively influences social entrepreneurship orientation directly. (2) Mindfulness upbringing perception indeed positively influence social entrepreneurship orientation partly through the mediating effect of prosocial motivation. (3) Moreover, findings suggest that perceived pressure from external stakeholders negatively moderates not only the relationship between prosocial motivation and social entrepreneurship orientation but also the overall mediation model. This demonstrates that social entrepreneurs with low-level perceived pressure from external stakeholders improve their social entrepreneurship orientation quickly when they have high prosocial levels. Based on these findings, the researchers conclude that social entrepreneurship orientation may be achieved more effectively through the complex process of mindfulness upbringing perception, prosocial motivation and perceived pressure from external stakeholders. (4) External stakeholder pressure has a restraining effect on the positive effect of mindfulness upbringing perception and public welfare orientation. Under a high external stakeholder pressure, the promotion effect of mindfulness upbringing perception on public-welfare orientation will be significantly inhibited. Compared with public-welfare orientation, external stakeholder pressure has a weaker negative moderating effect on the positive relationship between mindfulness upbringing perception and competitive orientation. In other words, external stakeholder pressure has a more significant negative impact on the public-welfare orientation. Under high stakeholder pressure, social entrepreneurs will suffer more significant damage to the public welfare orientation of social entrepreneurship, while competition orientation will be less negatively affected.

## Data availability statement

The datasets presented in this study can be found in online repositories. The names of the repository/repositories and accession number(s) can be found in the article/supplementary material.

## Ethics statement

The studies involving human participants were reviewed and approved by the Nanjing University of Posts and Telecommunications. The participants provided their written informed consent to participate in this study.

## Author contributions

TS contributed to developing the theoretical framework and overall writing of the manuscript. XT contributed to data collection, data analysis, and editing. All authors contributed to the article and approved the submitted version.

## Funding

This article was supported by the following research funds: National Social Science Foundation of China (20CRK004).

## Conflict of interest

The authors declare that the research was conducted in the absence of any commercial or financial relationships that could be construed as a potential conflict of interest.

## Publisher’s note

All claims expressed in this article are solely those of the authors and do not necessarily represent those of their affiliated organizations, or those of the publisher, the editors and the reviewers. Any product that may be evaluated in this article, or claim that may be made by its manufacturer, is not guaranteed or endorsed by the publisher.

## Appendix 1 旋轉元件矩陣^a^

**Table:**

	元件				
	1	2	3	4	5
**MINDFULNESS UPBRINGING PERCEPTION**	0.949				
MINDFULNESS UPBRINGING PERCEPTION 2	0.882				
MINDFULNESS UPBRINGING PERCEPTION 1	0.852				
MINDFULNESS UPBRINGING PERCEPTION 3	0.848				
MINDFULNESS UPBRINGING PERCEPTION 4	0.704				
MINDFULNESS UPBRINGING PERCEPTION 5	0.580				
**PROSOCIAL MOTIVATION**		0.947			
PROSOCIAL MOTIVATION 4		0.936			
PROSOCIAL MOTIVATION 1		0.802			
PROSOCIAL MOTIVATION 2		0.769			
PROSOCIAL MOTIVATION 3		0.742			
PROSOCIAL MOTIVATION 5		0.730			
**PERCEIVED PRESSURE FROM EXTERNAL STAKEHOLDERS**			0.890		
PERCEIVED PRESSURE FROM EXTERNAL STAKEHOLDERS 4			0.882		
PERCEIVED PRESSURE FROM EXTERNAL STAKEHOLDERS 3			0.789		
PERCEIVED PRESSURE FROM EXTERNAL STAKEHOLDERS 2			0.781		
PERCEIVED PRESSURE FROM EXTERNAL STAKEHOLDERS 1			0.738		
**PUBLIC-WELFARE ORIENTATION**				0.869	
PUBLIC-WELFARE ORIENTATION 1				0.755	
PUBLIC-WELFARE ORIENTATION 2				0.729	
PUBLIC-WELFARE ORIENTATION 3				0.676	
PUBLIC-WELFARE ORIENTATION 4				0.649	
PUBLIC-WELFARE ORIENTATION 5				0.592	
**COMPETITION ORIENTATION**					0.896
PUBLIC-WELFARE ORIENTATION 2					0.812
COMPETITION ORIENTATION 1					0.731
COMPETITION ORIENTATION 3					0.696

*擷取方法:主體元件分析。 轉軸方法:具有 Kaiser 正規化的最大變異法. a. 在 6 疊代中收斂循環。*
